# *In situ* degradation of dairy cattle feedstuffs using reusable local nylon fabric bags

**DOI:** 10.14202/vetworld.2022.2234-2243

**Published:** 2022-09-18

**Authors:** Despal Despal, Ouldya Fasya Alifianty, Adinda Putri Pratama, Fransiska Febrianti, Dwierra Evvyernie, Indah Wijayanti, Norma Nuraina, Indri Agustiyani, Annisa Rosmalia

**Affiliations:** 1 Department of Animal Nutrition and Feed Technology, Faculty of Animal Science, IPB University, Bogor, West Java, Indonesia; 2 Study Program Nutrition and Feed Technology, Department of Animal Nutrition and Feed Technology, Faculty of Animal Science, IPB University, Bogor, West Java, Indonesia; 3 Study Program Nutrition and Feed Science, Department of Animal Nutrition and Feed Technology, Faculty of Animal Science, IPB University, Bogor, West Java, Indonesia

**Keywords:** digestibility, feed quality, *in situ*, local fabrics, nylon bag

## Abstract

**Background and Aim:**

Disposable imported nylon bags used in an *in situ* digestibility measurement restrict the effort of scientists to obtain more accurate information about ruminant feed quality due to their low affordability and environmentally unfriendly characteristics. This study aimed to find reusable local nylon fabrics to substitute imports.

**Materials and Methods:**

Five local fabrics (B1 = Abutai, B2 = Taffeta, B3 = Organza, B4 = N57, and B5 = M100) were used to make nylon bags and compared with the imported bag (B6 = Ankom technology). The research consisted of three steps: (1) Observing the similarity of the local nylon bag’s hole number to the imported bag. (2) Testing feed *in situ* degradation (F1 = Napier grass, F2 = Cornmeal, and F3 = Dairy cattle total mixed ration) using bags B1–B6. (3) The reusability of the bag was tested using different washing methods (under running water [R1], rinse [R2], and ultrasonic water bath [R3]).

**Results:**

It was shown that the hole numbers of B1 (1223 hole numbers) and B2 (1245 hole numbers) were not significantly different from B6 (1248 hole numbers). It was shown with dry matter degradability measurement using the *in situ* method that there was no significant difference in feed solubility (a), potential degradability (b), and the effective degradation between local fabrics (B1–B5) and B6. According to the degradation rate (c), there were interactions between the feeds and fabrics. For F1, all local fabrics were similar to B6, while for F2, only B1 was different from B6. For F3, only B5 was different from B6. It was also shown by the organic matter degradability measurements that there was a similar trend. The exception was the solubility (a) component in F3, in which it was shown that B1 was also different from B6. It was shown in the reusable test that there was no difference in the weight of the bag before and after all washing methods. In contrast, the hole number increased due to the shrinking of the bag after drying in a 60°C oven.

**Conclusion:**

According to this *in situ* study, local nylon bag B2 can substitute imported bags. A lower drying temperature is suggested to prevent shrinking and make the bag reusable.

## Introduction

Ration formulation based on digestible nutrients information is more accurate than based on chemical composition [1] in estimating dairy cattle performance. Digestible nutrients are generated through several methods, including feeding trials (*in vivo*) [2], *in vitro* [3], and *in situ* [4]. Among these methods, *in vivo* is more accurate but highly variable, costly, and time-consuming [5]. In addition, this method requires large quantity of samples [2, 6]. The *in vitro* method can accurately estimate *in vivo* digestibility, requiring a small quantity of samples and measure the digestibility rapidly [7]. However, this method requires sophisticated laboratory equipment. Consequently, *in situ* can be the choice to determine feedstuff degradation. Orskov and McDonald [8] developed the *in situ* method and used different incubation times, enabling researchers to study the fermentation kinetics of feeds in the rumen [9, 10]. It was possible to study feed digestibility by incubating feed in the rumen using this method. The feed was placed in a nylon bag (*in sacco*) inside the rumen of a fistulated cow. Some advantages of the *in situ* method are considered less expensive [11], simple, quick, and reproducible [12]. In addition, such methods do not require chemicals. The *in situ* method can measure digestibility in a natural rumen environment [13]. Therefore, this method is more reliable in estimating *in vivo* organic matter digestibility and microbial protein synthesis [5].

Several factors influence the precision of feed digestibility measurement using the *in situ* method. Among such factors, it is possible to include digestion rate, bag porosity, preparation method, feed particle size, microbial contamination, rinsing method, incubation in different cows, days, bags, and chemical analysis of residues [13]. The material used to make the bag for the *in situ* methods includes the Dacron fabric [5, 14], polyester [15, 16] (ANKOM Co., Fairport, NY), polyamide [17], the blend of polyester and silk [4], and nytex (mononylon cloth #3-270-53 ASTM, B and SH Thompson and Co. Ltd., Montreal) [18] materials. Ankom technology produces the most frequent bags in the current *in situ* method. It is a disposal bag that uses a sealer to form the bag. It is unaffordable for most researchers in developing countries due to its price and complicated import procedures. The disposal bag is less environmentally friendly [19]. Bags used in the *in situ* methods should have several characteristics to avoid bias in measurement. The bag material should not be digested or interfere with microbial activity in the rumen. It should facilitate microbial movement in and out of the bag to digest the feed and release the digestion product. The bag should be sealed to prevent undigested feed particle loss from the bag [16]. Many researchers have tried to use local materials to make sample bags for the *in situ* method [4, 20]. Natural and synthetic fibers are produced in Indonesia. The country is also one of the largest synthetic fiber-producing nations, exporting about 130,000 tons of artificial fiber for yarn, staples, and textiles [21]. Fiber is used in the textile industry, primarily synthetic fiber, such as nylon and polyester [22]. Therefore, local fabric materials for *in situ* methods are available, such as abutai, taffeta, organza, N57, and M100. Fabric materials are commonly available in Indonesia and its neighboring countries. However, some limited tests have been conducted on local fabric materials used in making nylon bags for the *in situ* method.

Therefore, this study aimed to find local fabrics *in situ* bags that produce feed degradation kinetics similar to Ankom technology. The bag seal method using micro-nylon thread on the sewing machine was also tested for its reusability to improve environmental friendliness.

## Materials and Methods

### Ethical approval

The cannulation surgery of animals was carried out by a licensed veterinarian and followed the protocol for handling and care of animals, according to the IPB University Animal Ethics Committee.

### Study period and location

This study was conducted from January 2021 to March 2022 in the Field Laboratory of Dairy Nutrition Division, Faculty of Animal Science, IPB University, Indonesia.

### Hole number measurement and physical characteristics

Each type of the fabric (abutai [B1], taffeta [B2], organza [B3], N57 [B4], M100 [B5], and Ankom [B6]) was cut into 1 × 1 cm in size. Cuttings of the fabrics were observed under Nikon Eclipse E100 microscopes in triplicate with 40 ×. After that, the fabric hole was calculated manually. The physical characteristic image of the fabrics was produced using a smartphone (Samsung Note 10+, made in Korea) camera (16 megapixels) on the binocular surface.

### *In situ* study

The *in situ* study was conducted in the rumen of two fistulated bull Frisian Holstein (body weight [BW] ± 510 kg, 4 years old). The bulls were fed forage and concentrate with a 60:40 w/w dry matter (DM) forage to concentrate ratio. The daily DM requirement was calculated based on 2% BW. The nutrient content of the forage and concentrate is shown in Table-1 [23].

**Table-1:** Feed nutrient content of donor animal (fistulated bull) ration.

Feedstuff	DM	Ash	CP	EE	CF	NFE	TDN*
	
	(%)	% DM					
Napier grass	21.40	13.59	12.48	1.56	35.58	36.79	52.01
Concentrate	90.85	10.35	6.91	5.25	15.89	61.60	71.71

DM=Dry matter, CP=Crude protein, EE=Ether extract, CF=Crude fiber, NFE=Nitrogen-free extract, TDN=Total digestible nutrient.

* TDN=−14.8356 + 1.3310 (%CP) + 0.7923 (%NFE) + 0.9787 (%EE) + 0.5133 (%CF). Wardeh [23]

The experiment used a 6 × 3 factorial randomized block design. Factor 1 was the fabric type used in nylon bag making (B), while factor 2 was the feed type (F). The feed tested was forage (F1), cornmeal (F2), and total mixed ration (TMR) (F3). The sample for *in situ* studies was prepared by drying, grinding, and sieving the feeds to pass a 1 mm screen. Five grams samples (F1, F2, and F3) were put into nylon bags (B1, B2, B3, B4, B5, and B6), sealed using an impulse sealer, tied using cable ties, and incubated in the rumen of the fistulated bull for 0, 3, 6, 12, 24, 48, and 72 h. Each treatment was repeated 4 times. The size of the bags used was adjusted according to feed type. A 10 × 20 cm for F1, 5 × 10 cm for F2, and 7.5 × 15 cm for F3. The F3 was formulated according to the requirements of a high-producing lactating cow (BW = 445.21, milk production = 20.2 L/d, and milk fat = 3%). The nutrient content of the feeds tested is shown in Table-2 [23]. Before inserting bags into the fistulated rumen, the bags were grouped according to incubation time using a different color of plastic rope. After incubation, each group was removed by pulling out the distinctive color rope.

**Table-2:** Nutrient content of feed tested.

Feedstuff	DM	Ash	CP	EE	CF	TDN*
	
	(%)	% DM				
Napier grass	21.40	13.59	12.48	1.56	35.58	52.01
Cornmeal	91.82	0.93	10.29	3.56	2.06	73.47
TMR	88.57	11.72	12.63	4.45	10.20	59.90

DM=Dry matter, CP=Crude protein, EE=Ether extract, CF=Crude fiber, NFE=Nitrogen-free extract, TDN=Total digestible nutrient.

* TDN=−14.8356 + 1.3310 (%CP) + 0.7923 (%NFE) + 0.9787 (%EE) + 0.5133 (%CF). Wardeh [23]

Dry and organic matter degradation was measured after a certain incubation time in the rumen. The bags were cleaned using running water for about 5 min to remove rumen feed particles, liquids, and microbes. The washed nylon bag was placed in the oven at 60°C for about 7 days. Then, it was opened one by one to remove the feed sample residue. After that, the residue was then analyzed for its dry and organic matter according to the Association of Official Analytical Chemists [24]. Determining dry matter (DM) and organic matter (OM) degradations were referred to the exponential equation [8]: Pt = a + b(1-e^-ct^), where Pt = Fraction degraded at time t, a = The soluble fraction, b = Potential degradation, e = Natural log, c = The degradation rate of b component, and t = Incubation time. While, the effective DM degradability (effective degradation [ED]) was calculated using the equation: ED = a + (bc/[k+c]); a, b, and c are as described above and k is rumen outflow rate (k assumption 0.05 h^-1^ for F1 and 0.06 h^-1^ for F2 and F3).

### Reusable tested

The reusability of the nylon bag was tested visually by observing the condition of the bag. The weight and hole number changes after *in situ* degradation using three different washing systems (running water [R1], rinsed [R2], and ultrasonic water bath [R3]) were also observed. Visual observations were focused on bag damage, while weight and hole number changes were conducted by comparing the initial and final *in situ* incubation conditions. Visible conditions, bag weight, and hole number after incubation were measured after washing the used bag using three different washing systems and after the bags were dried in the oven at 60°C. The digital scale laboratory-type OHAUS PA214C (made in the USA) was used to measure the weight of the nylon bags, while the microscope-type Nikon Eclipse E100 was used to count the hole number.

### Statistical analysis

The data for hole number measurement before usage were analyzed using a one-way analysis of variance followed by Duncan’s multiple range test. The data from the *in situ* study were analyzed using a one-way analysis of variance by SPSS 20.0 version (IBM Corp., NY, USA). p < 0.05 was considered statistically significant. Duncan’s multiple range test was used to analyze the differences in the significant results. Then, the data of the reusable tests (weight and hole number changes) were analyzed using the paired t-test.

## Results

### Hole number

The similarity between local fabrics (B1, B2, B3, B4, and B5) and standard bags (B6) was observed from the hole numbers and microscopic images, as shown in Table-3. The statistical analysis showed that only B1 and B2 had hole numbers similar to those of B6. At the same time, B3, B4, and B5 had more hole numbers. Microscopic images of B1 and B2 also resembled those from B6.

**Table-3:** Hole number of various types of nylon bags on a microscope 4×10.

Nylon bag	Hole number	Figure	Nylon bag	Hole number	Figure
B1	1223±32^d^	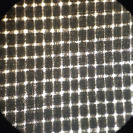	B4	6373±46^a^	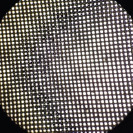
B2	1245±40^d^	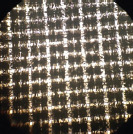	B5	1765±84^c^	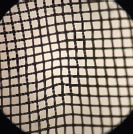
B3	4447±70^b^	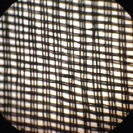	B6	1248±42^d^	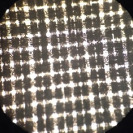

Different superscripts show significant different (p < 0.05). B1=Abutai, B2=Taffeta, B3=Organza, B4=N57, B5=M100, B6=Ankom

### *In situ* study

The DM degradation characteristics of the feed tested are shown in Table-4. It is shown in the table that b, a + b, and ED values were not significantly different among the nylon bags. Nevertheless, the values differed significantly between the feeds tested, where F3 was bigger than F1 and F2. The B6 (Ankom) solubility value (a) was not significantly different from the other bags (B1–B5). However, the solubility value (a) of B3 (13.24%) and B4 (14.26%) were significantly different from B5 (9.37%).

**Table-4:** DM degradation parameters on different feed and nylon bags.

Parameters	Feed	Nylon bags						Mean

		B1	B2	B3	B4	B5	B6	
a (%)	F1	10.34	10.80	12.38	12.81	11.31	10.44	11.34^b^
	F2	11.73	9.56	8.38	11.88	8.12	10.30	10.00^b^
	F3	14.21	15.52	20.88	19.35	8.46	13.37	15.30^a^
	Mean	11.90^ab^	11.63^ab^	13.24^a^	14.26^a^	9.37^b^	11.19^ab^	
b (%)	F1	28.90	28.03	36.73	40.01	37.96	30.67	33.72^b^
	F2	17.94	40.46	17.31	50.13	36.15	33.93	32.66^b^
	F3	57.63	55.06	70.85	55.31	54.25	74.09	61.20^a^
	Mean	32.75	39.92	38.98	47.86	41.75	43.70	
c (%/h)	F1	1.62^g^	2.30^g^	3.35^efg^	2.85^efg^	2.86^fg^	1.58^g^	2.43
	F2	19.0^a^	5.10^defg^	10.9^bc^	5.15^defg^	6.35^cdefg^	6.39^cdefg^	8.80
	F3	8.77^cde^	8.27^cdef^	4.20^defg^	9.22^cd^	15.2^ab^	7.40^cdefg^	8.84
	Mean	9.78	5.22	6.14	5.74	8.12	5.12	
a+b (%)	F1	29.73	38.83	49.11	52.81	49.27	41.12	43.48^b^
	F2	29.67	50.02	25.69	62.01	44.28	44.24	42.65^b^
	F3	71.83	70.58	91.74	74.66	62.71	87.46	76.50^a^
	Mean	41.19	51.56	52.22	62.12	51.12	54.89	
ED (%)	F1	16.99	18.82	26.49	22.17	25.43	17.14	21.17^c^
	F2	25.25	26.34	19.15	28.51	26.53	26.76	25.51^b^
	F3	48.18	47.35	44.61	49.03	45.12	54.26	48.09^a^
	Mean	28.50	29.53	28.76	31.80	31.20	30.76	

Means in the same row and column with different superscripts significantly different (p < 0.05). B1=Nylon bag abutai, B2=Taffeta, B3=Organza, B4=N57, B5=M100, B6=Ankom, F1=Napier grass, F2=Cornmeal, F3=TMR, a=The soluble fraction, b=Potential degradation, c=The degradation rate of b component, ED=DM degradability

The value of c ranged from 1.58% to 19.0%/h. There was an interaction between the bag type and the feeds tested. In F2, B1 (19.0%/h) was different from B6 (6.39%/h). At the same time, in F3, B5 (15.2%/h) was different from B6 (7.40%/h). Other bags and feeds tested produced a DM degradability rate (c) similar to the standard bag (B6).

Organic matter degradability characteristics are shown in Table-5. Coefficient b was not significantly influenced by the treatments. At the same time, a + b was influenced by the feeds tested only. The a + b value of F1 and F2 (42.72 % and 43.62 %, respectively) was significantly lower than F3 (76.91 %). The a and c coefficients show interactions between the treatments. The a values in F1 and F2 did not significantly differ between the bag types. Still, only B1 (22.29%) was similar to B6 in F3. The c coefficient was influenced by the interaction between the bag type and feed tested. There was no difference in the c coefficient between the bags type in F1. Only B1 coefficient (17.5%/h) was different from B6 (4.12%/h) in F2. At the same time, B4 (13.2%/h) and B5 (15.0%/h) were significantly different from B6 (3.97%/h) in F3. Both factors significantly influenced the ED values, but there was no interaction between them. Only the B4 (31.50%) bag type produced a significantly different result than B6 (22.06%). The ED values of F1 (13.90%) and F2 (18.03%) were significantly lower than F3 (49.87%).

**Table-5:** OM degradation parameters on different feed and nylon bags.

Parameters	Feed	Nylon bags						Mean

		B1	B2	B3	B4	B5	B6	
a (%)	F1	1.07^d^	1.06^d^	1.61^d^	3.82^d^	2.29^d^	1.08^d^	1.62
	F2	5.04^d^	3.74^d^	2.04^d^	2.95^d^	2.40^d^	3.11^d^	3.21
	F3	22.29^a^	9.16^c^	14.86^b^	13.73^b^	12.55^bc^	22.29^a^	20.08
	Mean	15.29	4.25	5.24	6.21	4.92	7.50	
b (%)	F1	33.67	33.89	38.64	57.89	47.64	34.87	41.10
	F2	19.61	52.23	18.93	55.27	51.25	45.14	40.40
	F3	37.33	59.42	76.68	59.65	51.57	56.32	56.83
	Mean	29.56	47.52	41.84	57.42	50,03	44.46	
c (%/h)	F1	1.07^f^	1.32^f^	2.86^ef^	2.53^f^	3.60^def^	1.47^f^	2.14
	F2	17.5^a^	4.23^def^	10.1^bcd^	6.40^def^	3.78^def^	4.12^def^	7.61
	F3	9.97^bcde^	8.37^cdef^	4.50^def^	13.2^abc^	15.0^ab^	3.97^def^	9.17
	Mean	9.36	4.64	5.81	7.39	7.46	3.18	
a+b (%)	F1	34.74	34.95	39.84	61.71	49.36	35.68	42.72^b^
	F2	24.65	55.98	20.97	58.22	53.64	48.25	43.62^b^
	F3	85.24	68.58	91.53	73.38	64.13	78.61	76.91^a^
	Mean	48.76	56.09	49.23	69.40	54.72	52.96	
ED (%)	F1	6.63	7.86	14.46	24.43	22.46	7.57	13.90^b^
	F2	18.65	17.83	11.14	21.22	19.66	19.70	18.03^b^
	F3	68.17	43.69	40.88	54.62	47.33	44.51	49.87^a^
	Mean	27.79^ab^	21.26^b^	20.46^b^	31.50^a^	28.22^ab^	22.06^b^	

Means in the same row and column with different superscripts significantly different (p < 0.05). B1=Nylon bag abutai, B2=Taffeta, B3=Organza, B4=N57, B5=M100, B6=Ankom, F1=Napier grass, F2=Cornmeal, F3=TMR, a=The soluble fraction, b=Potential degradation, c=The degradation rate of b component. ED=DM degradability

**Figure-1:**
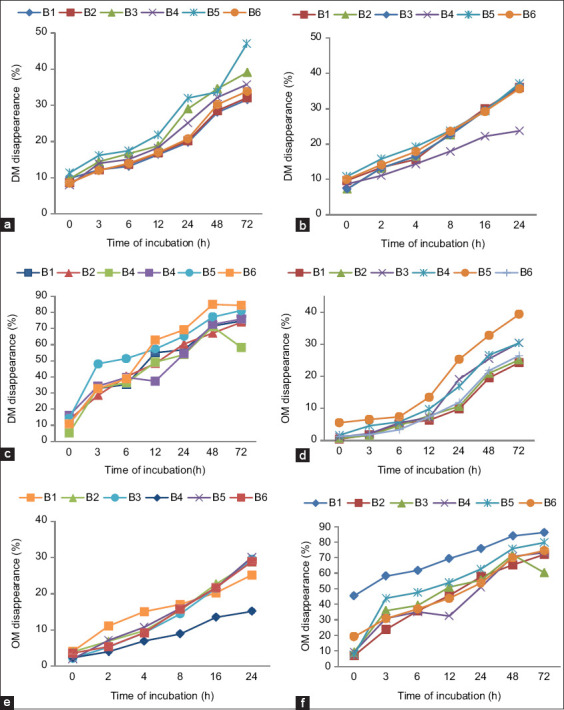
Dry matter disappearance of (a) F1, (b) F2, and (c) F3; organic matter disappearance of (d) F1, (e) F2, and (f) F3.

The DM and OM disappearance of feeds tested using different bag types during observations are shown in Figures-1a-f. The (a) graph shows an overlap curve between B1, B2, and B6, while B3, B4, and B5 were located above B6 at all observations. The (b) graph shows an overlap curve between all bag types except for B4. In (c) graph, until 6 h of observation, only the B5 curve lay above other curves. However, after 6 h, the TMR DM in B6 disappeared rapidly to reach a similar point with B5 at the incubation period of 72 h.

The OM disappearances of forage (d) followed a similar pattern to the (a) graph. In contrast, B1 and B4 produced a different pattern from B6 in cornmeal (e). The (f) graph showed a more varied disappearance of TMR between different bag types. The B1 and B5 curves were laid above the others. Nonetheless, B4 and B3 in specific hour observations were lower than B6.

### Reusable tested

The bags did not have any physical damage after the *in situ* study. The weight and hole changes of nylon bags after washing with different methods are shown in Tables-6 and 7. The weight of nylon bags before and after *in situ* using different washing systems had an insignificant result in almost all bags, except for B1 and B4 using the R2 washing system and B2 bags using the R3 washing system.

**Table-6:** Weight of nylon bags on different washing method.

Nylon bags	Washing method			Mean

	R1	R2	R3	
B1				
Before *in situ*	1.840 ± 0.050	1.820 ± 0.047	1.810 ± 0.074	1.823 ± 1.056
After *in situ*	1.850 ± 0.057	1.838 ± 0.048	1.840 ± 0.061	1.843 ± 0.052
t-test	0.097	0.001	0.403	0.090
B2				
Before *in situ*	1.226 ± 0.042	1.217 ± 0.048	1.217 ± 0.050	1.220 ± 0.044
After *in situ*	1.224 ± 0.037	1.207 ± 0.056	1.212 ± 0.050	1.215 ± 0.046
t-test	0.649	0.267	0.044	0.077
B3				
Before *in situ*	1.143 ± 0.055	1.148 ± 0.048	1.170 ± 0.052	1.176 ± 0.058
After *in situ*	1.394 ± 0.374	1.149 ± 0.086	1.166 ± 0.060	1.236 ± 0.240
t-test	0.172	0.218	0.514	0.341
B4				
Before *in situ*	1.268 ± 0.027	1.259 ± 0.019	1.261 ± 0.016	1.263 ± 0.020
After *in situ*	1.264 ± 0.037	1.262 ± 0.008	1.256 ± 0.008	1.261 ± 0.023
t-test	0.528	0.041	0.512	0.568
B5				
Before *in situ*	0.703 ± 0.015	0.667 ± 0.037	0.690 ± 0.017	0.687 ± 0.028
After *in situ*	0.704 ± 0.014	0.683 ± 0.022	0.588 ± 0.228	0.658 ± 0.135
t-test	0.155	0.292	0.349	0.408
B6				
Before *in situ*	1.469 ± 0.181	1.469 ± 0.181	1.569 ± 0.076	1.502 ± 0.153
After *in situ*	1.533 ± 0.038	1.470 ± 0.151	1.570 ± 0.065	1.524 ± 0.101
t-test	0.352	0.990	0.909	0.592

B1=Nylon bag abutai, B2=Taffeta, B3=Organza, B4=N57, B5=M100, B6=Ankom, R1=Under running water, R2=Rinse, R3=Ultrasonic water bath

**Table-7:** Hole number of nylon bags after washing on different methods.

Nylon bags	Washing method			Mean

	R1	R2	R3	
B1				
Before *in situ*	1202 ± 44	1194 ± 51	1192 ± 41	1202 ± 41
After *in situ*	1291 ± 60	1213 ± 37	1236 ± 38	1240 ± 60
t-test	0.008	0.544	0.045	0.023
B2				
Before *in situ*	1149 ± 27	1133 ± 41	1139 ± 50	1140 ± 39
After *in situ*	1238 ± 37	1264 ± 39	1227 ± 36	1243 ± 38
t-test	0.002	0.000	0.041	0.000
B3				
Before *in situ*	4156 ± 164	4147 ± 107	4148 ± 75	4150 ± 114
After *in situ*	4660 ± 66	4581 ± 130	4556 ± 22	4599 ± 92
t-test	0.000	0.001	0.000	0.000
B4				
Before *in situ*	7886 ± 148	7962 ± 170	7963 ± 179	7937 ± 161
After *in situ*	8722 ± 93	8851 ± 151	8757 ± 233	8777 ± 168
t-test	0.000	0.000	0.000	0.000
B5				
Before *in situ*	1407 ± 78	1400 ± 65	1413 ± 65	1407 ± 65
After *in situ*	1607 ± 64	1633 ± 60	1538 ± 81	1593 ± 77
t-test	0.005	0.001	0.065	0.000
B6				
Before *in situ*	944 ± 35	944 ± 42	943 ± 37	944 ± 36
After *in situ*	1083 ± 36	1040 ± 34	1087 ± 37	1070 ± 40
t-test	0.000	0.014	0.001	0.000

B1=Nylon bag abutai, B2=Taffeta, B3=Organza, B4=N57, B5=M100, B6=Ankom, R1=Under running water, R2=Rinse, R3=Ultrasonic water bath

The hole number of nylon bags before and after *in situ* in different washing systems showed a significant result in almost all bags. The exceptions were for B1 with the R2 washing system and B5 bags with the R3 washing system. The hole number of nylon bags after *in situ* treatment has increased.

## Discussion

### Hole number

As shown in Table-3, B1 and B2 have hole numbers and physical characteristics similar to B6. The B3 has almost identical fiber characteristics to B6 but a smaller thread diameter, resulting in a higher number of holes per cm^2^ and a larger pore size. The highest number of holes in B4 resulted from the smallest thread diameter and narrow distance between the threads, which resulted in a smaller pore size. In contrast to B4, the higher number of holes resulted from the small diameter of the thread with a broader distance between threads in B5, resulting in a larger pore size.

For *in situ* studies, the number of holes alone cannot explain the usability of fabrics to facilitate microbial movements. Fabrics of suitable quality should allow rumen microbes in and out to degrade the feed and flow freely the fermentation product out of the bags [16]. The current standard bags have 1248/cm^2^ hole numbers with thick thread, resulting in a small pore size. The company released a pore size of 53 with a standard deviation of ± 1.82 microns. The smaller the pore size of the fabrics, the less microbial movement, resulting in lower feed degradability. At the same time, the larger the pore size in higher feed degradability, the higher the undigested feed particle loss from the bags, and counted as a degradable part.

Depending on the feed particles tested, a pore size from 42 to 206 microns can still be used to facilitate the movement of ciliates [25]. Bender *et al*. [26] showed that a bag with 25 micron pore size resulted in a suitable model for predicting *in vivo* value. Combining bags with a small pore size (12 microns) with prolonged incubation is a good choice for measuring forage iNDF concentration [27]. The pore size used by Franzolin *et al*. [28] (50–100 microns) was in the range recommended by Michałowski *et al*. [25] and has been proven to facilitate ciliate movement. According to Czerkawski [29], bacteria sizes ranged from 0.6 to 2.3 microns, while protozoa ranged from 22 to 81 microns. Thus, the 42–206 micron pore size can still facilitate all the movements of bacteria and the majority of protozoa (*Entodinium simplex*, *Entodinium caudatum*, and *Entodinium dasytrica*) in and out of the bags freely, as required for the *in situ* studies [29].

In addition to the free movement of microbes, a bag used in an *in situ* study must prevent undigested feed loss from the bag [16]. Therefore, the particle size used must be larger than the pore size. Rahmat *et al*. [9] used a 5 mm forage particle size, while Rosmalia *et al*. [10] used 2 mm for concentrate. The 1 mm (1000 microns) feed particle used in the study was larger than the pore size of the fabric (50–100 microns), preventing undigested feeds from spilling out of the bags. The 1 mm particle size used in this study was based on Franzolin *et al*. [28], who reported that a 1 mm feed particle was better than 2 mm in DM, neutral detergent fiber (NDF), and acid detergent fiber (ADF) degradability. In addition, smaller particle sizes can increase the feed surface, which increases substrate exposure to microbes [30].

Table-3 shows the weak characteristics of the B3 thread, which may easily lead to damage during *in situ* studies. The risk of pore size change is high in such fabrics, leading to loss of undigested feeds from the bag and overestimated feed degradation. Therefore, based on the hole number and physical characteristics of the fabric, B1 and B2 can be alternatives to B6 used in an *in situ* study.

### *In situ* study

The insignificant value (a) produced from local fabrics compared to the standard bag (B6) showed that the pore size used was larger than the feed particle size. The soluble part (a) was feed particles smaller than the bag pore size. This can result from the grinding process or solubility in water. Using a laboratory mixer and sieved with a 1 mm screen, the grinding process produced less than a 1 mm feed particle size. A feed particle size less than the pore size left the bags without incubation in the rumen and counted as solubility. The fraction that disappears rapidly during incubation is known as solubility [8]. In this study, feed solubility ranged from 9.37% to 13.24%. The values were comparable to Guadayo *et al*. [4] for Napier grass in an *in situ* study with buffalos, but the cornmeal was lower than that reported by Ali *et al*. [31].

The soluble part of the feed mainly consists of sugar and starch [32]. Sugar is highly soluble in water [32], while starch depends on its type. Starch has a mainly semi-crystalline structure composed of amylopectin polymers and amylose [33]. Starch reduces its size after absorbing water and loses its stable crystalline structure during rinsing under running water. Sugar and starch contents in forage were lower than in concentrate [22], which showed a value of F1 smaller than F3. Although cornmeal is rich in starch, cornstarch is not soluble in cold water. Cornstarch mainly consists of linear and helical amylose and branched amylopectin [33]. Therefore, its solubility is similar to F1 and smaller than F3.

The b value represents the degradation of feed particles after incubation in the rumen [8]. Degradation of OM feeds by rumen microbes produces smaller molecules, such as volatile fatty acids and ammonia [32], which have a smaller molecular size and are, therefore, able to pass the bag pore size [16]. In this study, the degradation of feeds was not influenced by the bag type but by the feed tested. This means that the pore size of bags used in this study was in the range to facilitate microbial movement in and out of the bag, digest feeds, and release the product digestion but can prevent undigested feed loss from the bags.

The b values were different between the feeds tested, which were influenced by their nutrient content and characteristics. Forage such as Napier grass (F1) is rich in crude fiber (CF) [34]. It comprises hemicellulose, cellulose, lignin, and silica. The linkage of hemicellulose and cellulose to lignin makes it difficult for rumen microbe enzymes to digest CF [34]. Although F2 did not contain high CF, the lower b value of F2 was caused by the insolubility characteristics of its starch [33]. The feed should be soluble to be digested by rumen microbes. The rumen microbe in rumen fluid subsists on the soluble feed particle [35]. The insoluble characteristics of cornstarch caused the starch to produce clumping and floating on the rumen surface, lower contact with the rumen microbe, and be easily washed out from the rumen before it degraded. Such features result in lower rumen degradation [13]. The high b value in F3 resulted from the low CF and higher crude protein (CP) and ether extract (EE) of TMR. Fu *et al*. [6] showed that lowering fiber content (ADF and NDF) by adding some supplement in corn stalk and oat can increase OM and CP digestibility in an *in situ* study.

The c value was influenced by the interaction between the nylon bag type and the feed tested. Although almost all combination treatments produced similar c values to B6, the B1 in F2, B4, and B5 in F3 differed significantly from B6. The c value of B1 in F2 was larger than in B6, which showed that it degraded faster. However, due to its lower total degradability (a + b), the rapid degradation will stop and make the curve flat.

It was shown in the curve that in almost all feedstuff tested that the B2 bags produced a similar result to B6. This means that the B2 bags can be used as an alternative to the imported bag in ruminant feed degradability using an *in situ* study. In addition to its similar degradability pattern, similar results were also shown in the kinetics degradability (a, b, c, and ED). The similarity of the feed degradation curve and kinetics degradability was also supported by the physical resemblance between the fabrics of the two bags. These findings improved the accuracy of feeds quality evaluation by providing more affordable bags to evaluate feed degradability. Many researchers have tried to use local materials for routine *in situ* analysis. Figroid *et al*. [36] tested six nylon bags to estimate rumen utilization of grains. Two of the nylon bags used were local sources. It was shown in studies conducted by Valente *et al*. [20] and Kuwahara *et al*. [37] that F57 Ankom and non-woven textiles (NWT) have similar results. Ali *et al*. [38] also used nylon bags with pore size 37 from Nybolt, Zürich, Switzerland, to test grass silage degradation. From the physical and *in situ* utility characteristics, it can be concluded that B2 had similar results to B6.

### Reusable tested

Synthetic fibers, such as Dacron fabric [5, 14], polyester (ANKOM Co., Fairport, NY) [15], polyamide [17], the blend of polyester and silk [4], and nytex (mononylon cloth #3-270-53 ASTM, B and SH Thompson and Co. Ltd., Montreal) [18], have been used for bag fabrics in *in situ* studies. As one of the synthetic fiber producers, Indonesian local nylon fabrics have the potential to be used in the *in situ* study. The total amount of synthetic fiber exported by Indonesia was about 130,000 tons, and the total production was more than the national requirement [21]. The insignificant difference in weight before and after *in situ* conditions showed that rumen microbes did not digest the bags because the fabrics were not natural fibers. The synthetic fiber was preferable in the feed ruminal degradation study using the *in situ* method [11].

The indifferent weight before and after the *in situ* experiment and the absence of observed physical damage demonstrated the reusability of the bag. The F57 Ankom had less decreased tension of rupture (resistance) after ruminal incubation than nylon and NWT [20], making it possible for reuse after incubation [37]. The major problem with the disposable bag is the closure system using impulse sealer, which might break during handling and washing. This situation may be overcome by double sewn on all edges to prevent fraying [36], as used in this study.

Different washing systems used in this study were attempted to obtain cleaner reusable bags. However, insignificant differences in weight between the washing systems showed that all cleaning methods could be used. Although some researchers reported that the washing system influenced the bag clean [30, 39], all methods produced clean bags ready to be reused in this study. For safety considerations, the R3 method can be suggested in addition to R1. The R2 method was not recommended since it might damage the bags and potentially vary the fabric stretching after rinsing.

The increase in the hole number of the fabrics after the *in situ* study and similar bag weight showed that material shrinking might occur while drying the bags. This statement was also supported by Tian *et al*. [40]. For the reusability of the bag, it is necessary to measure the pore size of the used bag to guarantee that the size is larger than rumen microbes. Rumen microbes must move in and out of the bag to digest the feed [16, 20]. Adjusting the oven temperature during bag drying is also an alternative to prevent shrinking fabrics. Nocek [13] used 55°C for 48 h drying the nylon bag. Franzolin *et al*. [28] dried the bags in an oven at a temperature of 55°C for 72 h. A lower temperature for a more extended period can be tested.

## Conclusion

It is concluded that local nylon bags made from taffeta fabrics (B2) can substitute imported Ankom bags in an *in situ* study. A lower drying temperature is suggested to prevent shrinking and make the bag reusable.

## Authors’ Contributions

DD: Conceptualization and designed the study. DD, DE, AR, and IW: Supervised the experimental work. OFA, APP, FF, and IA: Conducted the experimental work. DD, IA, AR, and NN: Data interpretation, statistical study, collected literature, and drafted and edited the manuscript. DD, IW, and DE: Reviewed the manuscript. All authors have read and approved the final manuscript.
